# Cognitive Decline in Patients With Trigeminal Neuralgia: A Resting‐State fMRI Study

**DOI:** 10.1002/brb3.70434

**Published:** 2025-03-26

**Authors:** Xueyun Deng, Lihua Liu, Jiafei Chen, Zhi Liu, Hua Feng

**Affiliations:** ^1^ Department of Neurosurgery Southwest Hospital, Army Medical University Chongqing China; ^2^ Department of Neurosurgery Sichuan Provincial People's Hospital Eastern Sichuan Hospital & Dazhou First People's Hospital Dazhou Sichuan China; ^3^ Department of Neurosurgery The Affiliated Nanchong Central Hospital of North Sichuan Medical College Nanchong Sichuan China; ^4^ Department of Geriatrics Sichuan Provincial People's Hospital Eastern Sichuan Hospital & Dazhou First People's Hospital Dazhou Sichuan China; ^5^ Department of Radiology Southwest Hospital, Army Medical University Chongqing China

**Keywords:** cognition, cognitive decline, cognitive function, resting‐state fMRI, trigeminal neuralgia

## Abstract

**Objective:**

This study utilized functional magnetic resonance imaging (fMRI) data to investigate cognitive function changes in trigeminal neuralgia (TN) patients and healthy controls (HCs), and to elucidate the potential mechanism.

**Materials and Methods:**

The cognitive function of 34 patients with TN and 30 HCs was evaluated. Afterward, we calculated the amplitude of low‐frequency fluctuations (ALFFs), regional homogeneity (ReHo), and degree centrality (DC). These metrics were correlated with cognitive performance using the Spearman correlation analysis.

**Results:**

Patients with TN exhibited diminished cognitive performance compared to HCs. Increased mean ALFF (mALFF) levels were detected in the right temporal pole, superior temporal gyrus, and right insula in individuals with TN. These increases were negatively correlated with cognitive function. In contrast, decreased mALFF values were observed in the right lingual gyrus, bilateral calcarine, and left middle occipital gyrus, which were associated with improved cognitive function. Increased DC values were found in various areas, such as the right temporal pole, superior temporal gyrus, right opercular inferior frontal gyrus, bilateral medial superior frontal gyrus, left supplementary motor area, left anterior cingulum, and right middle cingulum in individuals with TN. These values negatively correlated with cognitive performance.

**Conclusion:**

TN patients exhibited impairments in multiple cognitive areas, such as attention, memory, executive function, visual perception and executive ability, information processing speed, and motor speed. The metrics ALFF and DC exhibited alterations in TN patients, suggesting that cognitive impairments may be linked to decreased functional activity in specific brain regions. Concurrently, certain cerebral regions may exhibit increased functional activity as a compensatory response to cognitive deficits. These findings hold significant theoretical value and clinical application potential, providing novel methodologies and perspectives for early diagnosis, personalized treatment, and efficacy evaluation. Such advancements are poised to enhance the overall treatment outcomes and quality of life for TN patients.

## Graphical abstract

This study identifies significant neural alterations involved in cognitive decline among trigeminal neuralgia patients using advanced fMRI techniques.

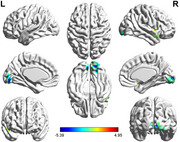

## 1 Introduction

Chronic pain poses a significant clinical challenge, with a global prevalence ranging from 1% to 76% (Cohen et al. 2021; Velly and Mohit 2018). It profoundly impacts patients' immune function, stress response, and cognitive abilities. The pathogenesis of chronic pain is intricate, the treatment process is time‐consuming, and the outcomes are often unsatisfactory. Persistent severe pain can influence brain networks associated with emotional arousal and cognitive regulation (Weng et al. 2017; Zhang et al. 2022). Studies indicate that a significant proportion of chronic pain patients experience cognitive disruptions. Research by Dick and Rashiq (2007) on 275 chronic pain patients revealed that 54% experienced multiple cognitive issues, including inattention, poor comprehension, slow reaction, disorientation, and mental confusion. Furthermore, Apkarian et al. (2005) demonstrated that pain stimulation activates various brain regions, including the insula, anterior middle cingulate gyrus, motor cortex, supplementary motor area, prefrontal cortex, and subcortical structures (basal ganglia, thalamus, and brainstem). This activation suggests a connection between pain and cognitive and motor brain areas, which impacts patients' cognition and behavior (Apkarian et al. 2005). Bagarinao et al. (2014) also highlighted the significant modulatory roles of the hippocampus and amygdala in emotion, memory, and pain‐related activities such as anxiety, depression, and aversion modulation. Researchers found that pain influences decision‐making ability and cognitive function in rats (Pais‐Vieira et al. 2009; Zhang et al. 2023). Particularly, trigeminal neuralgia (TN), with a prevalence of approximately 2 per 1000, involves chronic pain of the fifth cranial nerve and significantly affects patients' physical and mental health (DeSouza et al. 2016; Lee et al. 2021; Scholz et al. 2019). While neurovascular compression is commonly cited as a causative factor (Chen et al. 2022; Maarbjerg et al. 2015; Nurmikko and Eldridge 2001), recent studies have also identified structural and functional brain abnormalities in TN patients, though cognitive impacts are less well‐documented(DeSouza et al. 2016; Obermann et al. 2013; Tang et al. 2020; Tsai et al. 2018; Wang et al. 2017). For instance, Meskal et al. (2014) observed reduced cognitive performance in memory and attention among TN sufferers, and DeSouza et al. (2014) identified microstructural abnormalities in brain white matter. However, despite these findings, the specific relationship between cognitive decline and brain network alterations in TN patients remains inadequately explored, especially in terms of functional brain connectivity. Resting‐state fMRI (rs‐fMRI) is an imaging technique for noninvasively detecting whole‐brain function (Gao, Tong, et al. 2022; Gao et al. 2021; Gao, Xiong et al. 2022; Gao, Zhao, et al. 2022; Hang et al. 2022). ALFF represents the intensity of spontaneous neural activity, and ReHo indicates the synchronization of neural activity (X. Deng, Liu, Zhen, et al., 2022; Zang et al. 2007; Zang et al. 2004). Degree centrality (DC) describes the global characteristics between nodes based on their functional connectivity through graph theory. Recently, DC has been used to identify the core of brain networks, with increases indicating enhanced global connectivity and decreases indicating reduced whole‐brain connectivity. Scientists have employed DC to investigate a range of neuropsychiatric conditions, such as depression, psychosis, and manic depression (W. Deng et al. 2019; Guo et al. 2022; Yu et al. 2021; Zhou et al. 2019). However, the alterations in brain DC values in TN patients remain unclear. This study hypothesizes that cognitive impairment in TN patients is associated with changes in ALFF, ReHo, and DC in cerebral areas involved in cognitive function. Therefore, this study aims to pinpoint differential brain regions by calculating relevant metrics and analyze their correlation with cognition to reveal changes in brain function and reorganization. We have emphasized that while previous studies have explored brain network changes in chronic pain conditions, few have focused on TN, particularly in relation to cognitive decline. Moreover, there is a limited understanding of how TN‐related pain affects brain connectivity and how these changes might be linked to cognitive function. This study aims to fill this gap by investigating the brain network alterations in TN patients and exploring their potential relationship with cognitive decline, thereby contributing new insights to both the pain and neuroimaging literature

## 2 Materials and Methods

### 2.1 Inclusion/Exclusion Criteria

The criteria for inclusion were: experiencing pain on one side only; feeling typical sharp, intense, or stabbing paroxysmal pains from trigger areas; having no previous neurological or mental disorders or surgeries; and being right‐handed.

The exclusion criteria included: (1) craniocerebral trauma, intracranial tumor, cerebrovascular disease, encephalitis; (2) history of craniocerebral surgery; (3) cancer and other serious diseases; (4) other types of pain; (5) other conditions that might cause cognitive impairment.

Between December 2021 and June 2022, a total of 34 patients with primary TN were selected from the outpatient clinic at Southwest Hospital of Army Medical University. Patients experienced pain during the scanning process. Thirty right‐handed healthy controls (HCs) were also recruited. Clinical demographics are available in Table 1. Hospital Ethics Committee approved the study.

**TABLE 1:** A comparative analysis of demographic traits and cognitive abilities between trigeminal neuralgia (TN) patients and healthy controls (HCs).

	TN(*n =* 34)	HC(*n =* 30)	*Z*/*T* values	*p* value
Gender (male)	14.00 (41.2%)	13.00 (43.3%)	0.030	0.862^a^
Age (years)	57.00 (25.00)	54.50 (11.00)	−0.916	0.360^b^
Years of education (years)	5.50 (5.80)	8.50 (5.00)	−1.891	0.059^b^
Visual analog scale	7.33 ± 1.53	0	N/A	N/A
Duration	2.00 (3.56)	0	N/A	N/A
MoCA	18.50 (10.00)	25.00 (5.00)	−4.671	< 0.001^b***^
RAVLT	28.09 ± 14.51	41.40 ± 11.45	−4. 283	< 0.001^c***^
(Immediate recall)	3.00 (7.00)	8.50 (3.00)	−3.936	< 0.001^b***^
RAVLT	38.00 (34.00)	29.00 (9.00)	−3.347	0.001^b***^
(Delay recall)	50.00 (32.00)	41.00 (14.00)	−3.069	0.002^b**^
SCWT A (s)	110.41 ± 36.85	94.60 ± 29.62	2.145	0.036 ^c*^
SCWT B (s)	33.03 ± 18.62	42.57 ± 15.18	−2.475	0.016^c*^
SCWT C (s)	66.50 (49.00)	46.00 (27.00)	−2.974	0.003^b**^
SDMT	300.00 (165.00)	122.50 (153.00)	−2.677	0.007^b**^
HAMD	5.00 (8.00)	1.00 (3.00)	−4.552	< 0.001^b***^
HAMA	4.00 (7.00)	2.00 (2.00)	−4.199	< 0.001^b***^

*Note*: *Z* and *T* values were derived from nonparametric tests and *t*‐tests, respectively. Data were presented as mean ± SD, number (percentage), or median (interquartile range).

Abbreviation: N/A, not available.

^a^ *p* values were obtained using the chi‐square test.

^b^ *p* values were obtained using the Mann–Whitney test.

^c^ *p* values were obtained using the *t*‐test.

^*^ *p* < 0.05.

^**^ *p* ≤ 0.01.

^***^ *p* ≤ 0.001.

Our sample size determination was grounded in rigorous methodological considerations. Drawing on prior research (Seymour et al. 2015), we estimated an effect size of Cohen's *d* = 0.8. To achieve robust statistical power, we aimed for a power level of 80%, which is widely accepted in scientific research (Lima et al. 2022; Luna‐Villouta et al. 2023; Suwaidi et al. 2023). We employed a standard significance threshold of *p* < 0.05. Using G*Power software (Franz et al. 2007) to calculate the required sample size with these parameters, it was recommended that each group should have a minimum of 26 participants. Our study exceeds these recommendations, with 34 participants in the TN group and 30 in the HCs group. This larger sample size enhances the statistical power and reliability of our findings, ensuring the robustness of our results.

Pain was assessed using a visual analogue scale. A score of zero indicated no pain, whereas a score of ten represented the most intense, unbearable pain.

All participants completed the MoCA; RAVLT; Stroop tests A, B, and C; SDMT; and TMT A and B (X. Deng, Liu, Luo, et al., 2022).

### 2.2 MRI Scanning Parameters

Siemens Trio Tim 3.0T scanner was utilized to obtain MRI data. The parameters for rs‐fMRI scanning were established with a repetition time of 2000 ms, an echo time of 30 ms, a field of view of 192 mm × 192 mm, an acquisition matrix of 64 × 64, a flip angle of 90°, a slice thickness of 3.0 mm, and a voxel size of 3.0 mm × 3.0 mm × 3.0 mm. Below are the T1 scan details: slice thickness of 1 mm, TR of 1700 ms, TE of 2.52 ms, scan matrix of 256 × 256, and voxel size of 1.0 mm × 1.0 mm × 1.0 mm.

### 2.3 Imaging Pre‐Processing

The Restplus software (http://restfmri.net/forum/RESTplusV1.2) was utilized for data preprocessing and the computation of ALFF, ReHo, and DC values. The initial steps in preprocessing involved removing the first ten images and correcting slice timing. This was followed by realignment to ensure that head movements were kept within ± 2.0 mm or ± 2.0°. Additional steps included spatial normalization, detrending, and regression of several covariates such as cerebrospinal fluid, white matter, and head motion parameters. The data were then filtered within a frequency range of 0.01–0.08 Hz and subsequently smoothed. During preprocessing, ALFF was obtained without filtering, while ReHo and DC values were calculated without smoothing. Mean ALFF (mALFF) and mean ReHo (mReHo) were calculated for subsequent analysis.

### 2.4 Statistical Analysis

Statistical analysis was conducted using SPSS 23.0 (IBM). Data from both groups were analyzed with *t*‐tests in cases where the data distribution was normal and variances were equal; otherwise, the Mann–Whitney test was employed. The *χ* ^2^ test was utilized to compare qualitative data. An independent‐sample *t*‐test was conducted using SPM12.0 to compare patients with TN and HCs. ALFF, ReHo, and DC values from differential cerebral areas were extracted, and Spearman correlation analysis was conducted to explore associations between clinical data and cognition. A *p* value below 0.05 was deemed to be statistically significant.

The SPM12.0 software package was employed to perform an independent‐sample *t*‐test for the patients with TN and HCs, based on controlling age, gender, and years of education. An adjusted cluster‐level test was conducted for statistical analysis of ALFF, ReHo, and DC was defined as *p* < 0.001 at the voxel level, and family‐wise error (FWE)‐corrected *p* value < 0.05 at the cluster level. Then, the ALFF, ReHo, and DC values of differential brain regions were extracted, and the Spearman correlation analysis was used to assess the correlations between metrics value, cognitive function, and clinical indicators. A *p* value < 0.05 was considered statistically significant.

## 3 Results

### 3.1 Result of Demographic and Clinical Characteristics Comparisons

The clinical characteristics and demographics are summarized in Table 1. No significant differences in age, gender, or education level were observed between TN patients and HCs (*p* > 0.05). However, TN patients performed significantly worse than HCs on the MoCA, Stroop, RAVLT, TMT, and SDMT (*p* < 0.05; see Tables 1 and 2). TN patients reported a pain score of 7.33 ± 1.53, indicating high pain levels during the imaging process, which likely reflects the substantial distress experienced in daily life and its potential impact on cognitive function.

**TABLE 2:** Comparison of MoCA between TN patients and HCs.

	TN(*n =* 34)	HC(*n =* 30)	*Z* value	*p* value
Visuospatial executive	2.50 (3.00)	4.00 (2.00)	−3.895	< 0.001^***^
Naming	3.00 (1.00)	3.00 (0.00)	−2.970	0.003^**^
Attention	5.00 (2.00)	6.00 (0.00)	−4.900	< 0.001^***^
Language	1.00 (1.00)	2.00 (1.00)	−4.751	< 0.001^***^
Language: sentence repetition	0.00 (1.00)	1.00 (1.00)	−4.501	< 0.001^***^
Language: fluency task	1.00 (1.00)	1.00 (0.00)	−2.863	0.004^**^
Abstract thinking	0.00 (1.00)	2.00 (0.00)	−4.317	< 0.001^***^
Delayed recall	1.00 (3.00)	2.00 (2.00)	−2.485	0.013^*^
Orientation	6.00 (0.00)	6.00 (0.00)	−1.925	0.054
MoCA scores	18.50 (10.00)	25.00 (5.00)	−4.671	< 0.001^***^

*Note*: *p* and *Z* values were obtained by Mann–Whitney U.

^*^ *p* < 0.05.

^**^ *p* ≤ 0.01.

^***^ *p* ≤ 0.001.

### 3.2 Comparisons of ALFF, ReHo, and DC Values Between Groups

**FIGURE 1:**
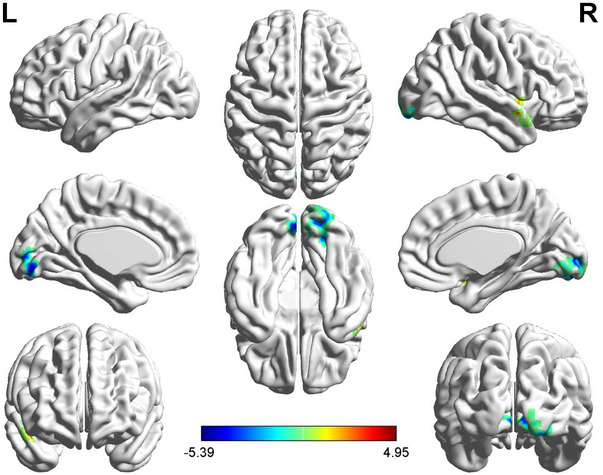
*T*‐values for brain regions with differential mALFF between the two groups. Compared to healthy controls (HCs), patients with trigeminal neuralgia (TN) exhibited increased mALFF in the right temporal pole, superior temporal gyrus, and insula. In contrast, TN patients exhibited reduced mALFF values in the right lingual gyrus, middle occipital gyrus, and bilateral calcarine regions. These findings highlight significant alterations in neural activity in specific regions associated with TN. The statistical significance level was set at *p*  < 0.001 at the voxel level, with a cluster‐wise family‐wise error (FWE) corrected *p*  < 0.05 threshold (40 voxels), ensuring robustness in the detected changes.

**FIGURE 2:**
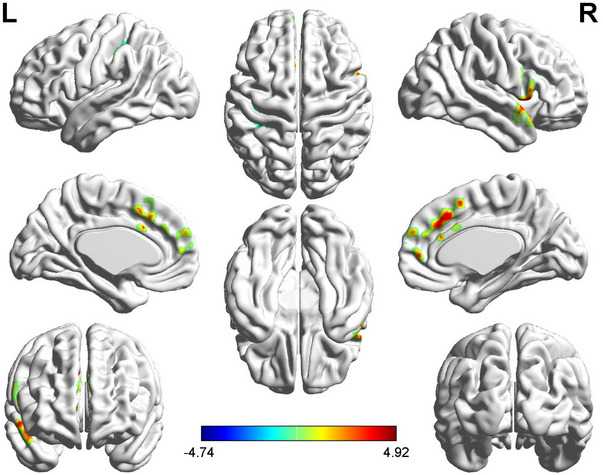
*T*‐values for brain regions with differential Degree Centrality (DC) between groups. Compared to the HCs, TN patients exhibited higher DC values in several brain regions, including the right temporal pole, superior temporal gyrus, bilateral medial superior frontal gyrus, right opercular inferior frontal gyrus, left supplementary motor area, left anterior cingulum, and right middle cingulum. Additionally, a reduction in DC values was observed in the left postcentral gyrus. These findings suggest altered functional connectivity in specific regions associated with TN. The statistical significance level was voxel‐wise *p* < 0.001 with cluster‐wise FWE corrected *p*  <  0.05 (73 voxels).

Patients with TN exhibited higher mALFF in the right temporal pole, superior temporal gyrus, and right insula compared to HCs, with lower mALFF values in the right lingual gyrus, left middle occipital gyrus, and bilateral calcarine. No significant changes in mReHo values were observed in TN patients. Patients with TN exhibited increased DC values in various brain regions including the right temporal pole, superior temporal gyrus, right opercular inferior frontal gyrus, bilateral medial superior frontal gyrus, left supplementary motor area, left anterior cingulum, and right middle cingulum, with decreased DC values observed in the left postcentral gyrus when compared to HCs (Figures 1 and 2).

### 3.3 Correlations of ALFF and DC With Cognition

The correlation between cognitive function and elevated mALFF values in the right temporal pole, superior temporal gyrus, and right insula of TN patients was demonstrated in Tables 3 and 4. Concurrently, reduced mALFF levels in the right lingual gyrus, left middle occipital gyrus, and bilateral calcarine showed a positive association with cognitive performance. Elevated DC values were detected in various regions of the brain in TN patients, including the right temporal pole, superior temporal gyrus, right opercular inferior frontal gyrus, bilateral medial superior frontal gyrus, left supplementary motor area, left anterior cingulum, and right middle cingulum. These values were found to have negative correlations with cognitive function, as illustrated in Table 5.

**TABLE 3:** Correlation analysis between mALFF and cognitive scale (1).

	Temporal_Pole_Sup_R mALFF (*n =* 64)	Insula_R mALFF (*n =* 64)
MoCA scores	−0.438 (*p* < 0.001)^***^	−0.296 (0.018)
Visuospatial executive	−0.359 (0.004)	−0.151 (0.234)
Naming	−0.161 (0.204)	−0.232 (0.066)
Attention	−0.455 (*p* < 0.001)^***^	−0.236 (0.06)
Language	−0.402 (*p* = 0.001)	−0.285 (0.022)
Language: Sentence repetition	−0.388 (0.002)	−0.223 (0.076)
Language: fluency task	−0.223 (0.079)	−0.294 (0.02)
Abstract thinking	−0.425 (*p* < 0.001)^***^	−0.285 (0.023)
Delayed recall	−0.234 (0.062)	−0.245 (0.051)
Orientation	−0.014 (0.913)	0.031 (0.805)
RAVLT immediate recall	−0.236 (0.061)	−0.221 (0.079)
RAVLT delay recall	−0.298 (0.017)	−0.209 (0.098)
SCWT A (s)	0.300 (0.017)	0.195 (0.125)
SCWT B (s)	0.282 (0.024)	0.279 (0.026)
SCWT C (s)	0.087 (0.492)	0.254 (0.043)
SDMT	−0.295 (0.018)	−0.225 (0.074)
TMT A (s)	0.283 (0.024)	0.274 (0.028)
TMT B (s)	0.365 (0.003)	0.389 (*p* = 0.001)

*Note*: Spearman's correlation coefficient (*r* value) and *p* value are shown.

Abbreviation: ALFF, amplitude of low‐frequency fluctuation.

^***^ *p* ≤ 0.001.

**TABLE 4:** Correlation analysis between mALFF and cognitive scale (2).

	Lingual_R_48 mALFF (*n =* 64)	Calcarine_L mALFF (*n =* 64)	Calcarine_R mALFF (*n =* 64)	Occipital_Mid_L mALFF (*n =* 64)
MoCA scores	0.315 (0.011)	0.394 (*p* = 0.001)	0.357 (0.004)	0.415 (*p* = 0.001)
Visuospatial executive	0.157 (0.216)	0.196 (0.120)	0.160 (0.208)	0.244 (0.052)
Naming	0.223 (0.077)	0.211 (0.094)	0.191 (0.132)	0.231 (0.066)
Attention	0.239 (0.057)	0.241 (0.055)	0.236 (0.061)	0.283 (0.024)
Language	0.273 (0.029)	0.354 (0.004)	0.283 (0.023)	0.373 (0.002)
Language: Sentence repetition	0.296 (0.018)	0.343 (0.005)	0.290 (0.020)	0.389 (*p* = 0.001)
Language: fluency task	0.016 (0.903)	0.207 (0.103)	0.097 (0.449)	0.147 (0.251)
Abstract thinking	0.318 (0.01)	0.355 (0.004)	0.341 (0.006)	0.456 (*p* < 0.001)^***^
Delayed recall	0.203 (0.108)	0.333 (0.007)	0.247 (0.049)	0.284 (0.023)
Orientation	0.063 (0.621)	0.157 (0.215)	0.129 (0.309)	0.122 (0.336)
RAVLT immediate recall	0.358 (0.004)	0.429 (*p* < 0.001)^***^	0.345 (0.005)	0.455 (*p* < 0.001)^***^
RAVLT delay recall	0.247 (0.049)	0.320 (0.010)	0.243 (0.053)	0.416 (*p* = 0.001)
SCWT A (s)	−0.258 (0.042)	−0.350 (0.005)	−0.295 (0.019)	−0.461 (*p* < 0.001)^***^
SCWT B (s)	−0.247 (0.049)	−0.312 (0.012)	−0.285 (0.023)	−0.412 (*p* = 0.001)
SCWT C (s)	−0.261 (0.037)	−0.289 (0.021)	−0.337 (0.006)	−0.402 (*p* = 0.001)
SDMT	0.243 (0.053)	0.294 (0.018)	0.285 (0.022)	0.424 (*p* < 0.001)^***^
TMT A (s)	−0.105 (0.408)	−0.209 (0.098)	−0.178 (0.160)	−0.337 (0.006)
TMT B (s)	−0.176 (0.163)	−0.252 (0.044)	−0.247 (0.049)	−0.512 (*p* < 0.001)^***^

*Note*: *r* value and *p* value are shown.

^***^ *p ≤* 0.001.

**TABLE 5:** Correlation analysis between DC value and cognition.

	Frontal_Inf_ Oper_R DC (*n =* 64)	Frontal_Sup_ Medial_L DC (*n =* 64)	Supp_Motor_ Area_L DC (*n =* 64)	Frontal_Sup_ Medial_R DC (*n =* 64)
MoCA scores	−0.335 (0.007)	−0.184 (0.145)	−0.207 (0.100)	−0.264 (0.035)
Visuospatial executive	−0.242 (0.054)	−0.139 (0.273)	−0.211 (0.094)	−0.181 (0.152)
Naming	−0.413 (*p* = 0.001)	−0.128 (0.312)	−0.178 (0.160)	−0.145 (0.254)
Attention	−0.344 (0.005)	−0.088 (0.490)	−0.285 (0.022)	−0.174 (0.170)
Language	−0.302 (0.015)	−0.097 (0.445)	−0.249 (0.047)	−0.160 (0.206)
Language: sentence repetition	−0.319 (0.010)	−0.082 (0.519)	−0.258 (0.039)	−0.168 (0.185)
Language: fluency task	−0.134 (0.296)	−0.144 (0.260)	−0.039 (0.760)	−0.126 (0.326)
Abstract thinking	−0.097 (0.445)	−0.327 (0.008)	−0.103 (0.417)	−0.446 (*p* < 0.001)^***^
Delayed recall	−0.130 (0.306)	0.020 (0.877)	−0.086 (0.500)	−0.128 (0.314)
Orientation	−0.042 (0.742)	−0.238 (0.059)	−0.126 (0.322)	−0.224 (0.076)
RAVLT immediate recall	−0.266 (0.034)	−0.224 (0.076)	−0.099 (0.438)	−0.295 (0.018)
RAVLT delay recall	−0.263 (0.036)	−0.332 (0.007)	−0.101 (0.426)	−0.373 (0.002)
SCWT A (s)	0.248 (0.050)	0.215 (0.090)	0.369 (0.003)	0.261 (0.039)
SCWT B (s)	0.187 (0.140)	0.298 (0.017)	0.288 (0.021)	0.260 (0.038)
SCWT C (s)	0.127 (0.319)	0.205 (0.104)	0.096 (0.452)	0.235 (0.062)
SDMT	−0.188 (0.136)	−0.241 (0.055)	−0.142 (0.265)	−0.289 (0.02)
TMT A (s)	0.129 (0.311)	0.168 (0.185)	0.153 (0.228)	0.229 (0.069)
TMT B (s)	0.209 (0.098)	0.215 (0.088)	0.244 (0.052)	0.228 (0.07)

*Note*: The *r* value and *p* value are shown.

Abbreviation: DC, degree centrality.

^***^ *p* ≤ 0.001.

## 4 Discussion

### 4.1 Cognitive Function of TN Patients

Individuals diagnosed with TN exhibited poorer performance on a variety of cognitive assessments, such as the MoCA, RAVLT, SDMT, Stroop, and TMT, suggesting a broad range of cognitive impairments in cognition such as memory, attention, executive function, visual perception and executive ability, information processing speed, and motor speed. This finding aligns with Meskal et al. (2014) who reported that TN patients had poorer performance in memory, attention, reaction time, and general cognitive function. The present study revealed that TN patients exhibited a pain score of 7.33 ± 1.53, consistent with the considerable impact of chronic pain on cognitive performance. Chronic pain has been established as a significant risk factor for cognitive impairments in various neuropsychological conditions (Meskal et al. 2014). In the case of TN, persistent and intense pain likely disrupts brain functional connectivity, particularly in regions such as the right temporal pole, superior temporal gyrus, and right insula, which are involved in emotional regulation and cognitive processes. These pain‐related changes in brain activity may explain the cognitive deficits observed in TN patients, affecting attention, memory, and executive function.

### 4.2 Correlations of ALFF, ReHo, and DC Metrics With Cognition

Understanding brain functions relies heavily on brain activity (Raichle 2010). ALFF and ReHo are reliable indicators with strong test‐retest consistency in rs‐fMRI studies (Zang et al. 2007, 2004). In 2007, Zang et al. introduced ALFF to represent local brain activity intensity (Zang et al. 2007). In 2004, Zang et al. proposed ReHo to indicate the synchronization of neural activity (Zang et al. 2004). DC, as an indicator of whole‐brain connectivity, reveals the core of brain networks by evaluating the functional connectivity between nodes and the entire brain through graph theory. Although numerous neuropsychiatric disorders have been studied using DC, no current studies have focused on DC in TN patients.

rs‐fMRI data were utilized to assess changes in brain function metrics among TN patients, examining correlations with cognitive performance. Results indicated changes in mALFF and DC metrics in TN patients.TN patients exhibited increased mALFF in the right temporal pole, superior temporal gyrus, and insula compared to HCs, indicating enhanced intrinsic brain activity and possible compensatory functions. On the other hand, reduced mALFF in the right lingual gyrus, middle occipital gyrus, and bilateral calcarine indicated weakened spontaneous activity in those regions. Elevated mALFF in the right temporal pole, superior temporal gyrus, and right insula showed a negative correlation with cognitive performance. The temporal pole and superior temporal gyrus, part of the medial temporal lobe, are crucial for memory (Shan et al. 2022; Stasenko et al. 2022). The insula regulates the generation and origin of sensations and emotions and acts as a switch between cognitive function‐related networks (Seeley et al. 2007; Sidlauskaite et al. 2016). Cognitive decline may lead to compensatory neural activity in these regions. Decreased mALFF in the right lingual gyrus, left middle occipital gyrus, and bilateral calcarine exhibited strong associations with cognitive performance. The lingual gyrus processes visual signals and is involved in complex image analysis and visual memory storage. The visual cortex at the lingual gyrus relates to vocabulary cognition, while the calcarine is associated with visual function, calculation, and logical thinking (Zhao et al. 2013). Reduced ALFF in these regions in TN patients may lead to corresponding cognitive dysfunction. Negative correlations were found between DC values in specific brain regions and cognitive performance, specifically in tasks assessing memory, attention, executive control, working memory, and motor speed (Savitz and Jansen 2003; Van Schependom et al. 2014). The temporal lobe, frontal lobe, supplementary motor area, and limbic system (cingulate gyrus) are related to RAVLT, SDMT, and Stroop and are closely associated with cognitive decline. Cognitive decline in TN patients may lead to a compensatory increase in DC values in these brain regions. No significant changes in ReHo values indicate that TN primarily affects functional differentiation and key node functions rather than overall brain function integration.

These findings provide new research directions for future studies on brain function in TN patients.

### 4.3 Significance of the Study

The study discovered that the decline in cognitive function in TN patients is closely associated with functional activity changes in specific brain regions. This provides new theoretical support for understanding the complex relationship between TN and cognitive functions. Early diagnosis of cognitive impairments in TN patients can be achieved through the use of fMRI and functional activity monitoring in specific brain regions, facilitating the foundation for early clinical interventions. A deeper understanding of the functional alterations in these specific areas enables the formulation of tailored therapeutic strategies, such as targeted rehabilitation or pharmacological treatments directed at regions exhibiting abnormal activity. Furthermore, employing metrics like ALFF and DC provides an objective method to assess changes in brain function pre‐ and posttreatment, thereby evaluating therapeutic efficacy. These methodologies represent a significant advancement in clinical evaluations. Additionally, the insights gained from this research can guide clinicians in the proactive prevention and management of cognitive dysfunctions in TN patients, thereby diminishing their impact on daily activities and enhancing overall quality of life. This comprehensive approach underscores the importance of personalized medicine and could potentially set new standards in the clinical handling of TN.

### 4.4 Limitations

Several limitations should be noted. First, the potential influence of antiepileptic drugs on brain structure and function cannot be ruled out, which might be a significant confounder. Second, the results of the rs‐fMRI are considered preliminary because of the limited sample size and cross‐sectional study design. Long‐term studies with more TN patients are needed to clarify the association between structural and functional abnormalities. Additionally, cognitive changes in patients with TN may be influenced by various other factors, including anxiety and depression.

## 5 Conclusion

Cognitive function decreased in TN patients, particularly in attention, memory, executive function, visual perception and executive ability, information processing speed, and motor speed. ALFF and DC markers were altered in TN patients. Cognitive impairment may be associated with decreased functional activity in certain brain regions, while reduced cognitive function may activate functional activity in other regions as a compensatory mechanism. This study employed a multi‐index analytical approach to deeply investigate the cognitive function changes and their underlying mechanisms in TN patients, establishing the link between alterations in specific brain region activities and cognitive impairments. These findings hold significant theoretical value and clinical application potential, providing novel methodologies and perspectives for early diagnosis, personalized treatment, and efficacy evaluation. Such advancements are poised to enhance the overall treatment outcomes and quality of life for TN patients.

## Author Contributions

**Xueyun Deng**: conceptualization, data curation, investigation, methodology, software, visualization, writing – original draft, writing – review and editing. **Lihua Liu**: conceptualization, methodology, supervision, validation, visualization, writing – review and editing. **Jiafei Chen**: conceptualization, investigation, methodology, project administration, software, writing – review and editing. **Zhi Liu**: conceptualization, data curation, investigation, methodology, supervision, validation, writing – review and editing. **Hua Feng**: conceptualization, data curation, investigation, methodology, project administration, resources, software, supervision, validation, visualization, writing – review and editing.

## Ethics Statement

The study was conducted in accordance with the Declaration of Helsinki and approved by the Ethics Committee of the First Affiliated Hospital of Army Medical University (protocol code: (A) KY2023059).

## Consent

All participants in the study provided informed consent.

## Conflicts of Interest

The authors declare no conflicts of interest.

### Peer Review

The peer review history for this article is available at https://publons.com/publon/10.1002/brb3.70434

## Data Availability

The raw data supporting the conclusions of this article will be made available by the authors.
